# Editorial

**Published:** 1915-03

**Authors:** 


### EDITORIAL

The Business Office of the *Journal-Record* is 23 West Alabama Street.

The Editorial Office is 1014-15 Atlanta National Bank Building.

Address all Business Communications to *Journal-Record of Medicine*, 23 West Alabama Street.

Make remittances payable to *The Journal-Record of Medicine.*

On matters pertaining to the Editorial and Original Communications, address Edgar G. Ballenger, M. D., Atlanta, Ga.

Reprints of Original Articles will be furnished at cost price. Requests for the same should always be made in THE MANUSCRIPT.

We will present, postpaid, on request to each contributor of an original article, twenty (20) marked copies of *The Journal-Record of Medicine* containing such article.

### FAME IN MEDICINE.

If what men do lives after them or gets vitality during their lives, for that matter, the things done must be worth while on the one hand and impressively presented so as to secure critical attention on the other.

There never was a good country doctor who did not meet conditions in a way to make him famous if all the facts were known. The tremendous difficulties he has to encounter in every day practice are such as to appall a city bred man accustomed to the conveniences of urban conditions and to the sense of security he has in the nearness of good counsel. The country doctor has to do all alone all the things that are presented to him and he must do them well. The people know him and they are his people in a peculiar sense. With none but lay help he has done things that the hospitals have hesitated to begin, but he has no way to telling about it. He hesitates to talk about it when he meets his city confreres. His fame rests in the consciousness of work well done.

A similar thing is true of a section of the country and hitherto, the South has been nearly inarticulate in telling the world what it has done in medicine and hygiene. Sometimes the facts have gotten out privately and have been exploited by other men with more eager audiences and the originator has been deprived of his rights. It is hard for a man with a new thing to give full credit to the source of his information. If he has elaborated it at all, he feels that it is all his, and if he has been started by some one else, it is that other man's business to look out for himself.

In recent months there have appeared in The Journal several preliminary papers and other evidences of original work among Southern men. Mizell and Niles and Dorsey have done fine work in Pellagra and they are gradually hewing the way to the discovery of this disease in its entirety. They are on different tracks but all striving for the truth. Boynton shows for the first time the passage through a stenosed pylorus of an instrument seen by the fluorosfcope. Hoke and Hodgson demonstrate a new method in the surgery of hammer toe. Hensen of Jacksonville, shows us the way to cleanse small towns of malaria. If we can not drain the place because of lack of money, he shows how to cure the mosquitoes and make them harmless. Claude Smith continues his discoveries in Hookworm. Bass comes to tell us the last word in Pyorrhea Alveolaris and to prove the entire treatment. Derr is doing splendid work in portraying and interpreting the skiagrams of the trunk and head. There are doubtless others that do not instantly come to mind.

Credit should be given every man for his work and the claims should be made so that they can always be substantiated. There is a satisfaction in knowing that one's record is good. It helps over numberless difficulties and we want every man to have the fame that is due his efforts. The finest work and the most careful thought in medicine is here with us and we want the evidences of it to be unmistakable.

It is the Journal's function to record such things and it is its desire to meet every man who has done things and to see that he has a record of them made. A record that will stand prior to that of a man living in a more articulate locality who might have gotten hold of the idea.

We believe in the interchange of ideas and we know that the widest intercourse and exchange of experiences is essntial to the best work, but we can not understand why the South should ever be in the least subservient to any other locality in her intellectual achievements, medical or otherwise. Our large cities now give ample clinical material. Our men are of the righest grade of workers and thinkers. We have only to say so and our title to fame is secure; we have only to make the claim and to come into our own.

---

### THE DESIGN.

The statisticians tell us that autopsies show the presence of tuberculosis in so many bodies where it had not been expected and where it had not shown any clinical evidence, that they have concluded that almost everyone has this disease at some time or another.

The pathologists demonstrate with ease and speed that the mouth constantly harbors the bacteria of dangerous diseases such as Pneumonia and that children will carry Diphtheria virus for a long time. We are told that 95% of adults have Pyorrhea Alveolaris and that the toxins coming therefrom are an incubus of the gravest character.

We know that where any sizable group of people are kept together without opportunity for greater cleanliness than is really natural to them, typhoid and typhus fevers appear and take their tremendous toll. We are constantly the hosts of malignant organisms and we use much of our vitality in fighting them so as to keep alive.

Why? Has the answer even been given? There is vanity and vexation of spirit in many of the cant phrases that have been put forth to account for human woes.

The human mind has the habit of measuring things in terms of itself and then in the same attitude attempting to discern and describe things immensely beyond it. Thus we are told of a design in all nature and all life and of a super-wisdom that regulates things toward a perfect consummation of a plan beyond our comprehension. We become fatalists and subserviently follow the custom of enduring things because it seems we must or of interceding perfunctorily to have the plan and the design changed when they run counter to our ideas of common sense.

We run along on the idea of Alpha and Omega. We have the concept that because in our own experience everything has a beginning and ending, these conditions must exist everywhere beyond our experience. We ascribe our own minute limitations as characteristic of the illimitable and we attempt to measure infinity by our own attributes.

Our patients will solemnly tell us that because a tonsil was created or an appendix placed in accord with some design, they should be allowed to remain there. The extremists among them will not submit to operation until enough of scare has been thrown into their ideas as to do away with mental assumptions and bring them to facts. In many instances the patients die because of the philosophic principles of the friends and relatives that are antagonistic to any interference with the design as they have interpreted it.

And we let them do it. We assume a dignified attitude and say that the responsibility is theirs and we retire as quickly as we can, but whoever heard of a man bold enough to tell the truth about the matter or of one who would attempt to teach his people during their health of the fallacies of their ideas as to illness and disease?

So long as the ideas of design persist, that long shall we have an immense and unreasonable obstacle to overcome in the way of real medical progress and service.

The idea of "design" is human. As applied to man with his countless dangers and stresses from his very birth, yes, from his ancestry to the third and fourth generation, the design is a bungle and reverence for it is in the way of progress.

